# Towards a metadata scheme for the description of materials – the description of microstructures

**DOI:** 10.1080/14686996.2016.1194166

**Published:** 2016-07-29

**Authors:** Georg J. Schmitz, Bernd Böttger, Markus Apel, Janin Eiken, Gottfried Laschet, Ralph Altenfeld, Ralf Berger, Guillaume Boussinot, Alexandre Viardin

**Affiliations:** ^a^ MICRESS® group at Access e.V., Aachen, Germany

**Keywords:** Metadata, hierarchy, 3D microstructures, HDF5, interoperability, multiphase materials, nomenclature, ontology, simulation chains, multiscale modelling, 60 New topics/Others, 403 CALPHAD/Phase field methods, 404 Materials informatics/Genomics, 402 Multi-scale modeling, 100 Materials, 300 Processing/Synthesis and Recycling, 500 Characterization, 200 Applications

## Abstract

The property of any material is essentially determined by its microstructure. Numerical models are increasingly the focus of modern engineering as helpful tools for tailoring and optimization of custom-designed microstructures by suitable processing and alloy design. A huge variety of software tools is available to predict various microstructural aspects for different materials. In the general frame of an integrated computational materials engineering (ICME) approach, these microstructure models provide the link between models operating at the atomistic or electronic scales, and models operating on the macroscopic scale of the component and its processing. In view of an improved interoperability of all these different tools it is highly desirable to establish a standardized nomenclature and methodology for the exchange of microstructure data. The scope of this article is to provide a comprehensive system of metadata descriptors for the description of a 3D microstructure. The presented descriptors are limited to a mere geometric description of a static microstructure and have to be complemented by further descriptors, e.g. for properties, numerical representations, kinetic data, and others in the future. Further attributes to each descriptor, e.g. on data origin, data uncertainty, and data validity range are being defined in ongoing work. The proposed descriptors are intended to be independent of any specific numerical representation. The descriptors defined in this article may serve as a first basis for standardization and will simplify the data exchange between different numerical models, as well as promote the integration of experimental data into numerical models of microstructures. An HDF5 template data file for a simple, three phase Al-Cu microstructure being based on the defined descriptors complements this article.

## Graphical abstract

**Figure:**
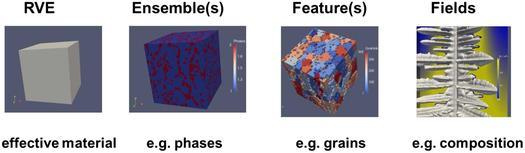

## 1. Introduction

Microstructures are one of the most important keys to understand materials. Microstructures are – besides the properties of the phases constituting the microstructure – one of the governing variables defining the properties of any material. There are numerous well-known approaches to extract properties of a technical material, either from only statistical values like the Hall–Petch relation correlating yield stress and average grain size,[1,2] or – more recent – from spatially resolved microstructures. Examples of the latter are the determination of flow curves based on numerical tensile tests,[3] the determination of the permeability of mushy zones,[4] the hot tearing susceptibility of steel grades,[5] the hardening and recrystallization during rolling processes,[6] and the crack initiation and crack propagation under mechanical load,[7] to name only a few.

Any microstructure is determined by the entire history of processing conditions during the manufacture of the material, starting from a well-defined initial condition of a homogeneous, isotropic and stress free liquid.[8] Microstructures are increasingly the focus of modern engineering, as they can be influenced and even be tailored by suitable processing schemes and dedicated alloy design.

While microstructures were historically recorded as 2D metallographic sections on glossy prints, current computational infrastructures allow for storage and retrieval of spatially resolved digital 3D (and even 4D) microstructure descriptions. Microstructures may originate from experiments, from simulations or may have been artificially created as synthetic microstructures.[9,10] The needs and the benefits of exchanging microstructure data between these different areas has recently been pointed out [11] and an HDF5 type data structure [12] has been identified as a pragmatic approach for a standardized, file based information exchange.[13] A missing link towards a seamless exchange of microstructure information remains the specification of a unified set of metadata descriptors allowing naming of the different entities in an HDF5 file describing a microstructure. The scope of the present paper is to provide a basic list of such descriptors and the reasoning leading to its specification.

**Figure 1.**
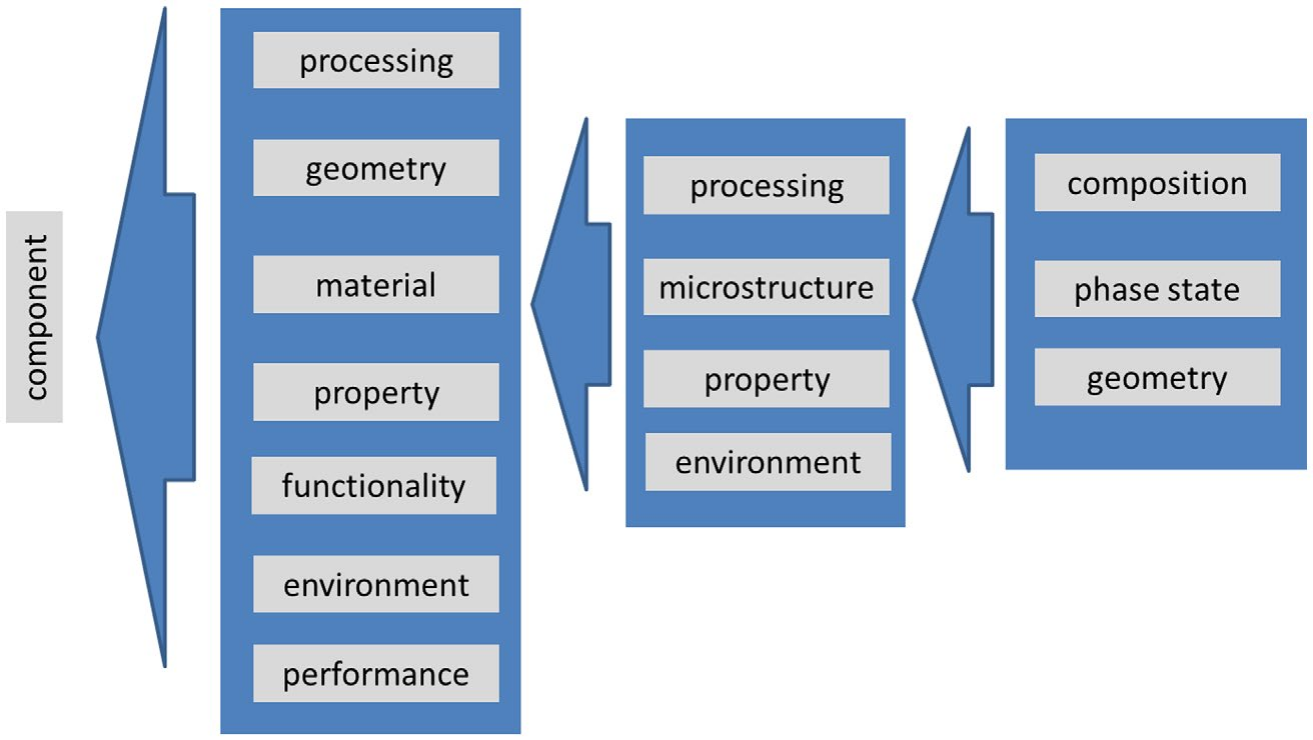
The four core ontologies for the description of materials (middle) as ingredients of any component (left column), adapted from [16, 17]. The term ‘substance’ in [17] is replaced by ‘microstructure’ for the current purpose. The term ‘performance’ indicating the evolution of properties under environmental/operational conditions has been added for the component. The present paper aims at providing a detailed and comprehensive description of substances/microstructures (right column) as a part of the description of a material (middle column).

‘Metadata’ are defined as ‘data about data’.[14] Metadata provide information that allows categorization, classification and structuring of data. In the area of materials modelling, metadata are meaningful, e.g. for physics models, numerical representations, solvers, workflows, processes, materials, properties, costs, and many others. In particular, metadata for materials play an important role as materials determine the properties, the functionality and eventually the performance of any component. Metadata for microstructures represent a subset of a much more comprehensive materials ontology, see e.g. [15–17], which specifies following four core ontologies: substance/material, process, property, and environment (Figure 1).

Microstructure models provide the link between models operating at the electronic, atomistic, and mesoscopic scales as depicted e.g. in [18], and models and tools operating on the scale of a component and its processing. A comprehensive description and a common understanding of the terminology being used to describe a – digital – microstructure thus is most important in view of an easy exchange of information and improved interoperability of a heterogeneous variety of software tools being available to describe various aspects of materials in an integrated computational materials engineering (ICME) approach.[19]

It seems important to note that materials and their microstructure in general undergo an evolution during their processing and in some cases also during their operation. This evolution may comprise phase changes, which often go along with a discontinuous change in the properties of the material. Such phase changes may be beneficial, e.g. in phase change materials for latent heat energy storage [20] or for computer memory applications.[21] Phase changes may also be detrimental, e.g. in the case of corrosion.[22]

**Figure 2.**
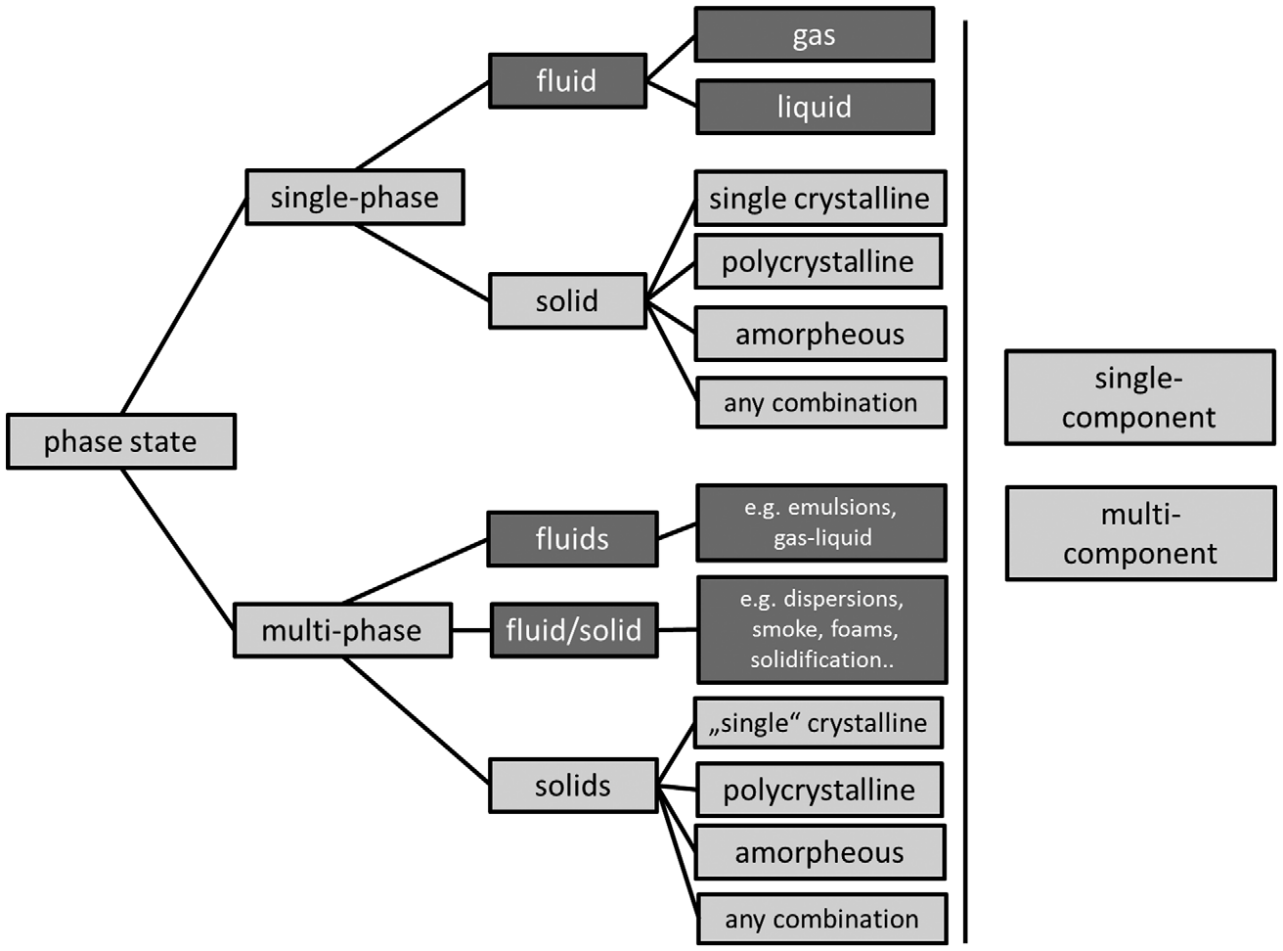
Structure classifying the phase state of a material. Any of the depicted phase states can occur in multicomponent systems (i.e. systems comprising multiple chemical elements). Single component systems (i.e. pure elements) only occur as single phase materials except at critical points, where e.g. solid and liquid phases may coexist only in a very narrow temperature range. Multiphase single crystals are to be understood as a single crystalline matrix containing secondary phases. The present article focuses on solids only (light gray) but the concepts can readily be extended to fluids (dark gray). This structure provides the basic and generic concept of classifying materials according to their phase state as detailed in the text.

Any complete metadata description of a microstructure thus has to provide the option to describe all phases possibly occurring in a material with a given chemical composition (Figure 2). The scope of the present article is to provide a minimum set of basic metadata descriptors.

Once defined, this set of basic descriptors provides a path for establishing relations between these descriptors to describe other entities. All these descriptors can then be applied to describe data generated by numerical models, by numerical simulations or data extracted from an experimental characterization of real materials. The set of descriptors thus will promote a seamless integration of experimental data into microstructure simulations and into materials models used in simulations at different scales.

**Figure 3.**
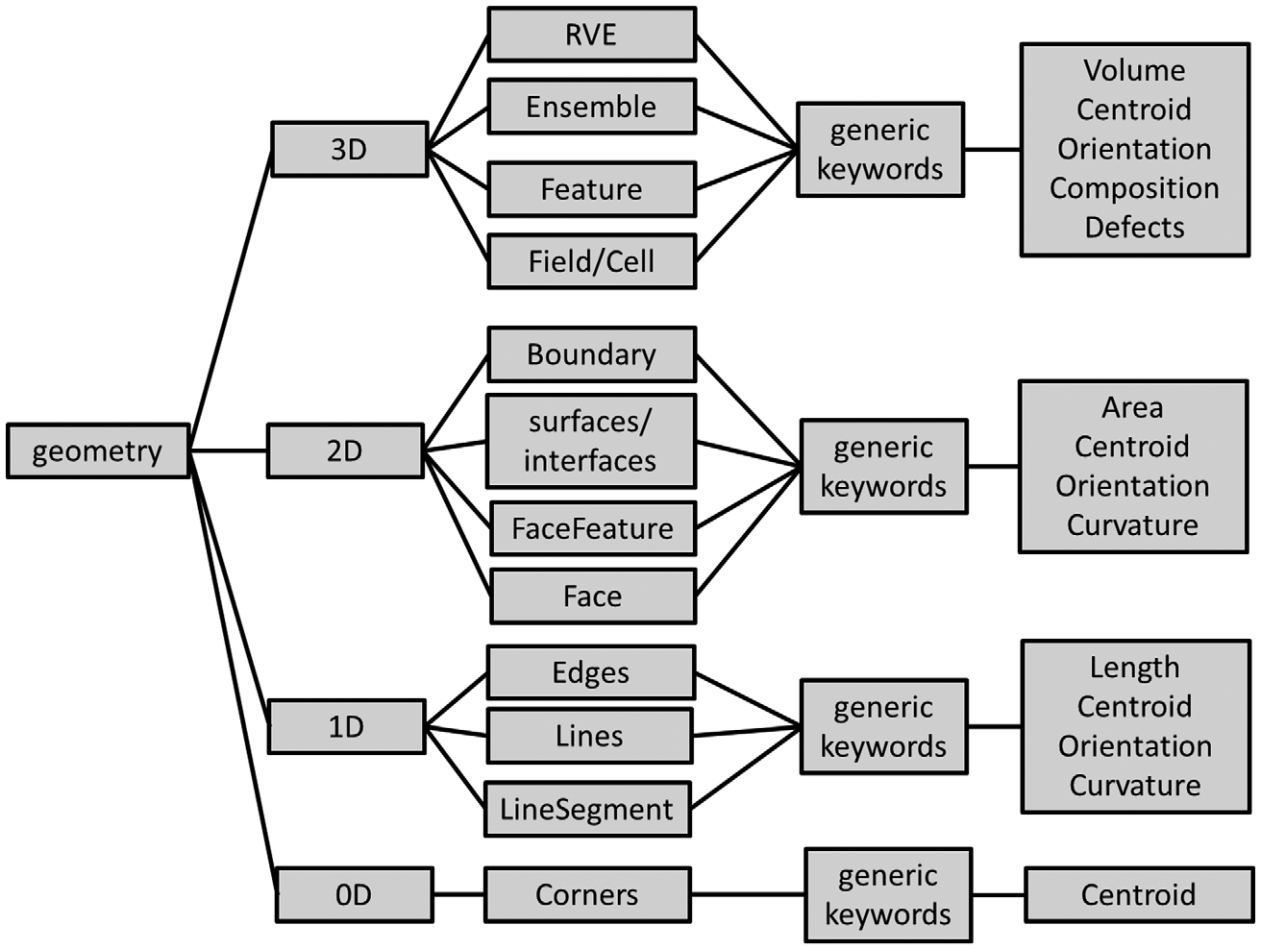
Dimensional hierarchy of the description of the geometry of a microstructure. Each dimension group has different subsets, which correspond to different levels of detail. The RVE in the 3D description, for example, provides average values and statistical information, while Field/Cell corresponds to the highest resolution. See text for further details and explanations of the terms in the boxes.

This article only discusses metadata descriptors describing a *static 3D microstructure picture at a given instant in time.* Adding any dynamics and kinetics will have to happen in future activities by adding further, separate descriptor lists. The description of the microstructures in this article is further limited to the mere geometric description of an arrangement of features in space (Figure 3). Aspects related to ‘allocation of properties’ to these different features are part of ongoing and future developments.

What is a material? Any material is ‘a number of atoms arranged in a volume’, where the number of atoms may be very large, and they will typically belong to several chemical elements. These atoms may ‘self-arrange’ and form *features* and *ensembles* of features like molecules, particles, precipitates, crystals/grains, different phases/crystal structures, defect structures, and eventually multiphase, polycrystalline materials.

The volume must be large enough to host the number of atoms and should be large enough to be representative for the heterogeneous material under consideration, thus becoming a ‘*representative volume element*’ (RVE).

Although basically all descriptors depicted in this article can probably also be used for atomistic arrangements (where each atom could be a ‘feature’), all further discussion is based on a continuum perspective with the features being grains, precipitates, pores, etc.

A material as seen from a continuum perspective not resolving any individual atoms thus becomes ’a number of features in an RVE’. These features in general interact via *fields*, e.g. stress fields, temperature fields, and magnetic fields. Fields are continuously defined in real space and thus are continuous functions of the position (x,y,z) and may also be functions of time. The distribution of a large number of discrete objects in a volume can also be described by a continuous field like the ’concentration field’ of atoms of a specific element. Any continuous *field* has to be discretized into *numerical cells* or *numerical elements* in order to make it accessible to numerical methods.

**Figure 4.**
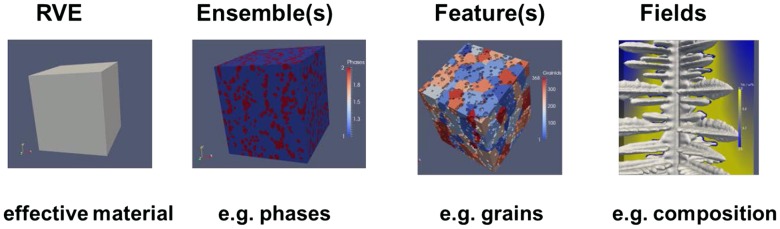
Hierarchical structure of materials.

Overall materials thus reveal a hierarchical structure at different levels as explained by the words in italics in the section above (Figure 4).

These different hierarchical levels will be discussed in the following sections: RVE (section 2.1); Ensemble (section 2.2); Feature (section 2.3); and Fields (section 2.4).

It seems important to note that the geometrical distribution of any feature or ensemble inside the RVE is fully determined by the highest resolved spatial information, which is available in ‘Fields’, as described in section 2.4.

A similar hierarchy also holds for 2D features of surface and interface data, from the smallest surface element, named a face, to ensembles of interfaces, e.g. all interfaces between different phases in a system or the entire surface/boundary of the RVE. These 2D features will be treated from small to large in section 3 according to the following scheme. This reverse method of description has been selected for reasons of didactic simplicity: Faces (sections 3.1 and 3.2); FaceFeature (section 3.3); Surface and Interfaces (section 3.4); RVE Boundaries (section 3.5).

The descriptors are sorted by following the above inherent hierarchy of complex microstructures which is largely defined by the different constituents and the corresponding length scales.

We propose a notation for the descriptors according to the following rules:

• Each descriptor starts with a capital letter.
• Any descriptor may be composed of different constituent specifiers, e.g. NumberAtoms or NumberMoles without blanks. Each constituent specifier starts with a capital letter again. Typical constituent specifiers are ‘Number’, ‘ID’, ‘Name’, ‘Type’ and others. Basically there is no limit for the number of constituent specifiers.
• Some entities can be specified as *descriptor relations* (see section 5), which are typically denoted by an underscore ‘_’ . An example is the descriptor relation Volume_Fraction.
• Descriptors followed by brackets ‘(ExampleID)’ are vector components. An example is AtomPercent(ChemicalElementID). In case of derived descriptors the brackets will always be located at the end of the descriptors, e.g. Volume_Fraction(ChemicalElementID).
• Descriptors are valid in both singular and plural forms, e.g. ‘FeatureID’ and also ‘FeatureIDs’. Plural is denoted by adding an ‘s’ at the end of the descriptor.
• Even if not explicitly stated in the present article all descriptors defined for one specific hierarchical level are, wherever this seems meaningful, also valid descriptors at any other hierarchical level, i.e. at the RVE, Ensemble, Feature and Field levels.

Section 5 will depict a scheme to derive descriptor relations based on the basic set of descriptors defined in sections 2, 3 and 4. Section 5 also provides details about a specific implementation of the proposed descriptors in a specific file format being based on HDF5. This template file is meant as a starting point for the further improvement of interoperability between numerous models drawing on microstructures. Besides the descriptors themselves this template file also already defines some attributes to the descriptors, e.g. data types, units, and array sizes.

Most explanations in the present paper – without any restriction to the generality of the descriptors – are discussed on a simple example of a binary alloy (Al-Cu) revealing two solid phases (alpha and theta) and one liquid phase, described on a simple voxel type grid.

## 2. Volumetric data

All data are in SI units unless explicitly specified. The units can be individually assigned as specific attributes to the different descriptors (see section 5.3). The units by default are specified for the entire volume and hold for all its subsystem (ensembles, features, cells).

### 2.1. RVE

#### 2.1.1. RVE geometry

**Figure 5.**
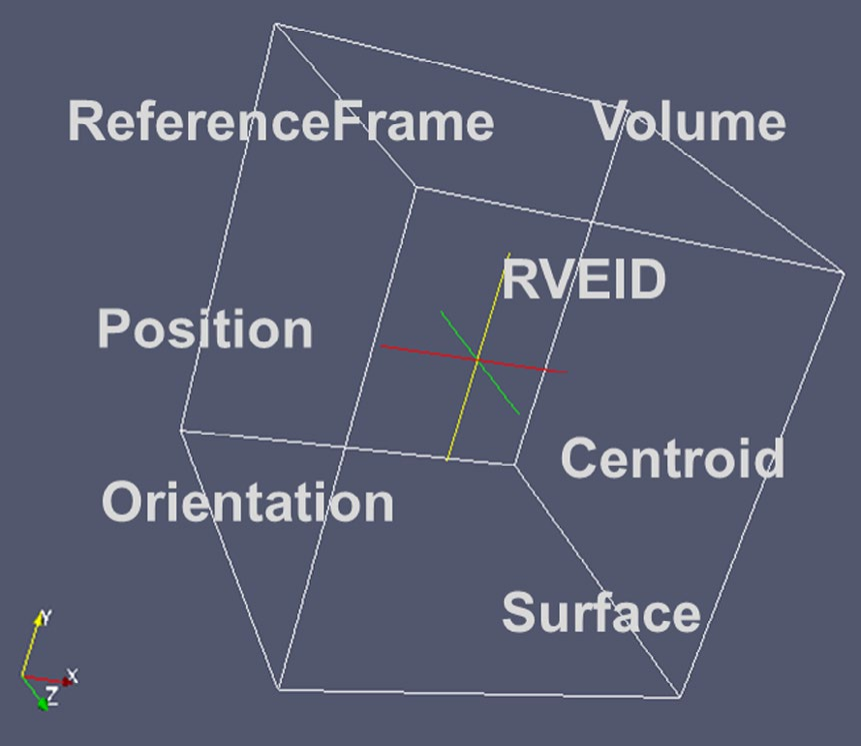
Illustration of the basic descriptors for the geometry of an RVE.

Microstructures by nature are related to an arrangement of features in space. Thus first there is a need to define the space being spanned to contain these features. This volume – if representative for the material – is called the representative volume element (RVE). The definition of the term RVE here follows the specification of an RVE in continuum mechanics,[23] where the RVE size has to be sufficiently large to be representative for a given property under consideration. The descriptors however are defined for any volume. A basic set of descriptors for any arbitrary volume in space is given in Figure 5. For extra details about the geometric description of the RVE please refer to sections 2.4 and 3.

##### 2.1.1.1. ReferenceFrameID

Describing the position of the RVE in space requires the specification of a reference frame. For the RVE this in general will be the ComponentFrame, i.e. the structural axis system of the component. For all features within the RVE, the frame of reference will be the RVE frame itself (Table 1).

**Table 1.** Specification of the different frames of reference. Only the RVE requires the specification of this ReferenceFrame. Larger scale frames may become interesting e.g. for logistics.

ReferenceFrameName	ReferenceFrameID
Unspecified	0
GPSSystem	1
FactorySystem	2
BuildingSystem	3
MachineSystem	4
ComponentFrame	5
RVEFrame	6
CrystalLattice	7
Cell system	8

##### 2.1.1.2. RVEID

Specifies the identifier for this particular RVE in a larger scale process simulation; allows information to be retrieved from this particular RVE and it to be distinguished from other RVEs.

##### 2.1.1.3. Position

Vector specifying the position of the Centroid of the RVE in the ComponentFrame, which is the default ReferenceFrame for the RVE. Other ReferenceFrames may be selected according to the ReferenceFrameID. The components of this vector are: PositionX, PositionY, PositionZ.

##### 2.1.1.4. Centroid

Vector specifying the position of the Centroid of the RVE in the RVEFrame. Components are: CentroidX, CentroidY, CentroidZ being described in the RVEFrame.

##### 2.1.1.5. Origin

Vector specifying the origin of the RVE. Components are: OriginX, OriginY, OriginZ. ReferenceFrame for the Origin is the Component Frame. In the RVEFrame OriginX, OriginY, OriginZ are all identical 0 by definition.

##### 2.1.1.6. Orientation(OrientationTypeID) or Orientation(OrientationTypeName)

Specifies the orientation with respect to the ReferenceFrame. OrientationTypeName and/or OrientationTypeID details the way orientation is described. The ReferenceFrame for the RVE by default is the ComponentFrame (Table 2).

**Table 2.** OrientationTypeName and OrientationTypeID.

OrientationTypeName	OrientationTypeID
Angle2D	1
EulerAngles	2
MillerIndices	3
Quaternions	4
AngleAxis	5

##### 2.1.1.7. Volume

Specifies the total volume of the RVE. The volume may result from an analytical expression like *a* ^3^ for a cube with size *a* or 4/3 𝜋*r* ^3^ for a sphere with radius *r* or from summation/integration of the volumes of all NumericalElements or NumericalCells into which the RVE is discretized.

##### 2.1.1.8. Surface

Specifies the total surface of the RVE.

#### 2.1.2. RVE composition

**Figure 6.**
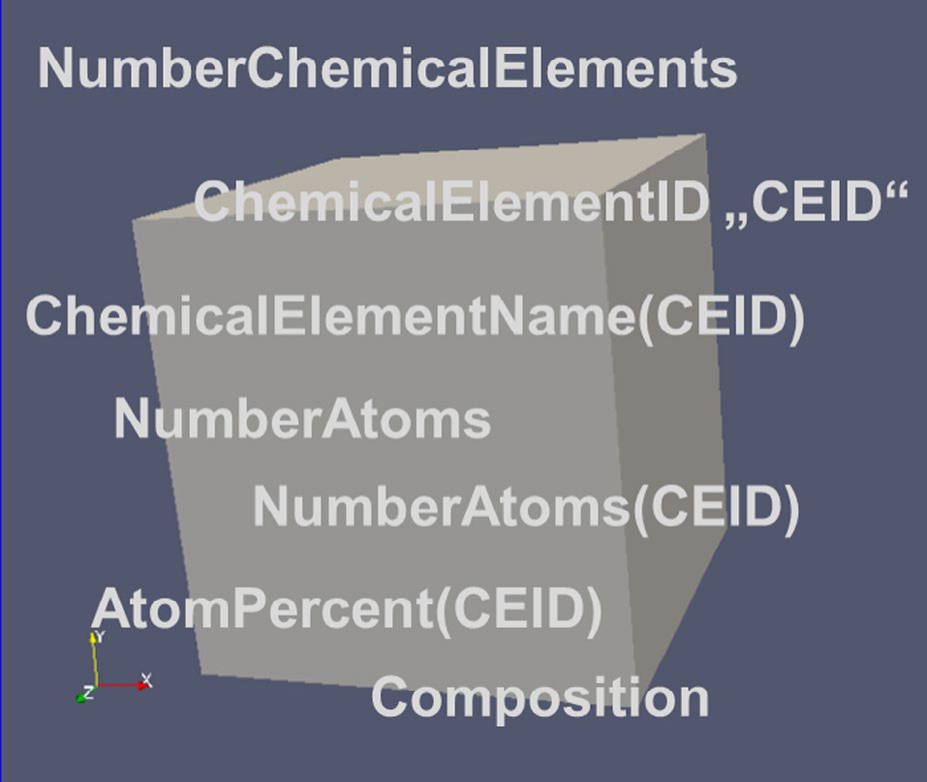
The major descriptors for the composition of an RVE.

As any material is ‘a number of atoms arranged in a volume’, where the atoms may be atoms of different chemical elements, a first set of descriptors is required to describe the composition of the material arises naturally (Figure 6).

##### 2.1.2.1. NumberChemicalElements

Corresponds to the number of chemical elements specified in the RVE. Examples for chemical elements would be Al and Cu in a binary alloy. The NumberChemicalElements would intuitively be 2 for this example. However, unspecified elements further increase the NumberChemicalElements by 1 and the ChemicalElementName ‘Ue’ (Unspecified element) has to be assigned to them to conform to the standard two letter nomenclature for chemical elements. In case no unspecified elements are present in the system, their NumberAtoms respectively NumberMoles for ‘Ue’ has to be set to 0.

##### 2.1.2.2. ChemicalElementID

Corresponds to a local ID for the chemical element. Runs from 0 to NumberChemicalElements. The ChemicalElementID (CEID) corresponds to the index of the vectors for ChemicalElementName, NumberAtoms, AtomPercent, etc. The CEID value 0 is exclusively used for unspecified elements ‘Ue’.

##### 2.1.2.3. ChemicalElementName(CEID)

Specifies the name of the chemical element with CEID. Names are specified according to the standard nomenclature in the periodic table of the elements, e.g. ‘Al’ for Aluminum, or ‘Cu’ for copper. Unspecified chemical elements are summarized under the ChemicalElementName ‘Ue’ (Unspecified element) and the CEID with value 0 is always used, whether present in the system or not.

##### 2.1.2.4. NumberAtoms

Specifies the total number of atoms (all elements) in the RVE. Used for small systems with few atoms only. For large systems it is given in moles. The distinction between mere integers for numbers and real values for moles will proceed via descriptor attributes (see section 5.3). For example, NumberAtoms (type=integer, units=1) for small numbers of atoms versus NumberAtoms(type=real, units=moles) for large numbers of atoms. It will be identical to 0 for unspecified chemical elements if no unspecified chemical elements are present.

##### 2.1.2.5. NumberAtoms(CEID)

Specifies the total number of atoms of the element with CEID in the RVE. Used for small systems with few atoms only. For large systems (moles) see attributes for units as specified above for NumberAtoms. It will be 0 for unspecified chemical elements if no unspecified chemical elements are present.

##### 2.1.2.6. NumberMoles_Fraction(CEID)

The NumberMoles(CEID)_Fraction is a *descriptor relation* (see section 5) and inserted here to account for typical applications in engineering. The NumberMoles(CEID)_Fraction of an element with CEID is defined as:

NumberMoles(CEID)_Fraction=NumberAtoms(CEID)/NumberAtoms

Please note that the ‘units’ and ‘type’ attributes of NumberAtoms(CEID) and NumberAtoms have to match in this case.

##### 2.1.2.7. AtomPercent(CEID) and MassPercent(CEID)

The AtomPercent of a chemical element with CEID is defined as:

$$\text{AtomPercent(CEID)}=100^{}*\text{NumberMoles}\_\text{Fraction(CEID)}$$

It is not a descriptor within the basic set, but a *descriptor relation*. It is introduced here to account for the common way of specifying the composition of a material. Another typical way to specify the composition is the use of mass_% of the individual alloy elements. Note that the introduction of a ‘MassPercent’ of an element with CEID goes beyond the mere geometric and enumeration specifications as depicted in this article. It already assigns an attribute/a property – namely their molar mass – to the atoms. The calculation of MassPercent follows the known rules and is not further detailed here in spite of introducing the descriptor itself.

##### 2.1.2.8. Composition(unit)

Composition(AtomPercent) and Composition(MassPercent) are vectors describing the relative abundance of the different chemical elements in a given system. They are specified via a ‘unit’ attribute (see section 5.3) in either AtomPercent (unit=at.%) or in MassPercent (unit=wt.%), respectively. The dimension of these vectors corresponds to the NumberChemicalElements (respectively NumberIsotopes in special cases). The individual components of the vector are given as AtomPercent(CEID) (respectively MassPercent(CEID)) each with values ranging from 0–100%. The sum of all composition vector components yields 100%. The ‘lowerbound’ and ‘upperbound’ can become part of the descriptor attributes (see section 5.3) and may be used to check consistency of the data.

Often also the term ‘concentration’ is erroneously – but widely – used to describe composition. In a strict sense it is a derived descriptor and refers to the number density of a chemical element, i.e.:

NumberAtoms(CEID)/Volume respectivelyNumberMoles(CEID)/Volume

where ‘Volume’ corresponds to the Volume of the RVE here. The formal descriptor relation for ‘concentration’ would be NumberAtoms_Density(CEID).

Some further descriptors refer to special cases when isotopes are present in the RVE:

##### 2.1.2.9. NumberIsotopes

Specifies the number of isotopes being present in the RVE. Equals NumberChemicalElements when isotopes are not explicitly considered. The same rules apply to unspecified isotopes as to unspecified elements.

##### 2.1.2.10. IsotopeID

Specifies the local IsotopeID for each isotope in the RVE. Runs from 0 to NumberIsotopes. Equals CEID when no isotopes are explicitly considered. The same rules apply to unspecified isotopes as to unspecified elements.

##### 2.1.2.11. IsotopeName(IsotopeID)

Specifies the name of the isotope with IsotopeID. ‘Names’ will be given as spinors (integer) in terms of number protons (Z) and number of nucleons (=protons+neutrons) (N): example IsotopeName=(Z,N). For unspecified isotopes: IsotopeName=(0,0)

#### 2.1.3. Ensembles in the RVE

Ensembles are defined as a number (or moles) of atoms being arranged in a defined way. Ensembles can be either discrete constituents (e.g. molecules) or continuous crystal structures or lattices of atoms with characteristic properties (e.g. phases). In a future advanced definition of ensembles ‘effective phases’, i.e. superstructures of multiple phases, can also be treated as ensembles. An example for an effective phase is pearlite, a superstructure of ferrite and cementite phases.

##### 2.1.3.1. NumberConstituents

Indicates the number of constituents in the RVE. Examples for constituents would be OH–, H+, H_2_O, etc. in water, or N_2_, O_2_, CO_2_ in air. Single atoms of H or O would be treated as constituents as well when opting for the constituent type description in order to assure the sum of all constituents to represent the entire system. The NumberConstituents may be larger than the NumberChemicalElements. The same rules apply to unspecified constituents as to unspecified elements.

##### 2.1.3.2. ConstituentID

Specifies the local ConstituentID for each constituent in the RVE. Runs from 0 to NumberConstituents. The same rules apply to unspecified constituents as to unspecified elements.

##### 2.1.3.3. ConstituentName(ConstituentID)

Specifies the name of the Constituent with ID ‘ConstituentID’. Names are still to be standardized. Names will be character strings e.g. ‘OH–‘, ‘H+’, ‘H_2_O’

**Figure 7.**
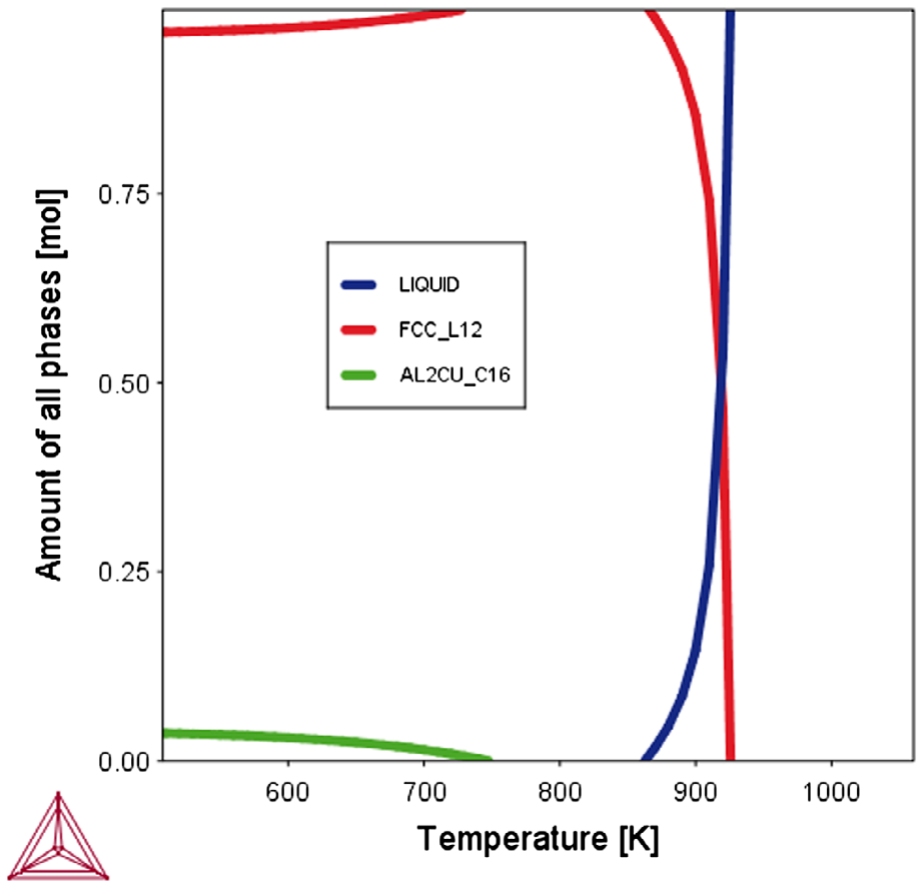
Equilibrium Volume_Fractions of different phases in an RVE with a defined overall composition can be calculated for given conditions (e.g. temperature, pressure). There is no information about the spatial distribution of these phases. Shown is the example of the α, Θ and Liquid phases in a binary Al-Cu alloy.

Knowing about the composition of the RVE either in terms of the amounts of chemical elements or the amounts of constituents implies the notion of phases which may or may not appear in this particular RVE (Figure 7).

**Figure 8.**
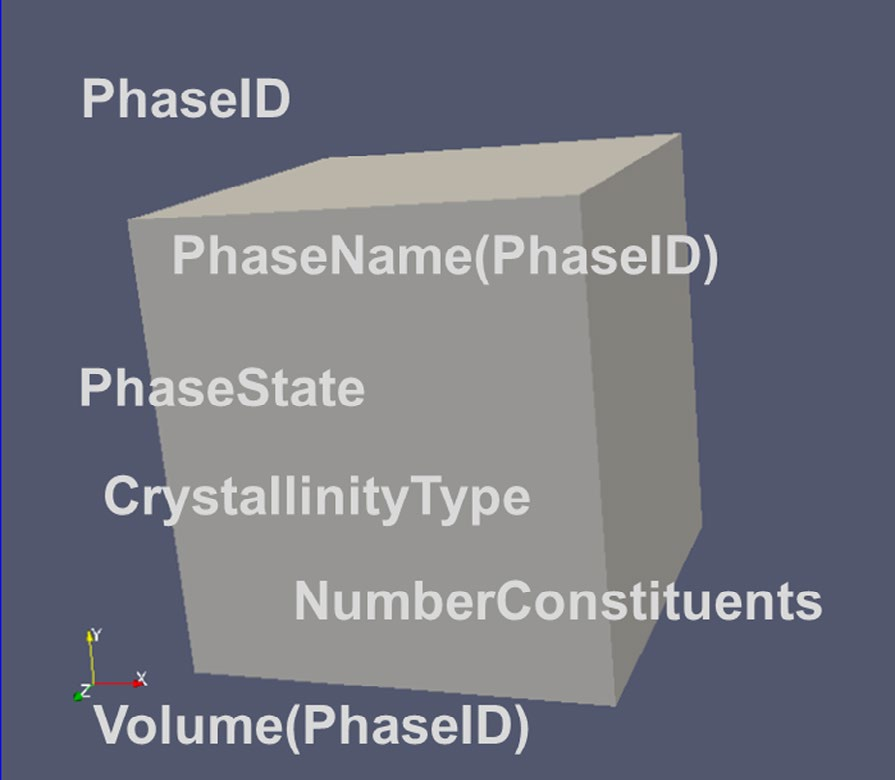
Major descriptors for the phases being present in an RVE.

Descriptors describing the presence of ensembles of atoms (i.e. the phases) are depicted in Figure 8. Note that there is no information about the spatial distribution of the phases associated with the notion about their possible existence for given conditions. Descriptors describing attributes of the ensembles are given in section 2.2.

##### 2.1.3.4. NumberPhases

Corresponds to the number of all phases present in the RVE. Basically it is possible to assign also phases actually not yet being present in the system, but which might form during its further evolution. For the static microstructures considered here such phases are assigned with a Volume being equal to 0.

##### 2.1.3.5. PhaseID

Specifies the local PhaseID for each phase in the RVE. Runs from 1 to NumberPhases. The value of NumberPhases is assigned with the PhaseID = 0

##### 2.1.3.6. PhaseName(PhaseID)

Specifies the name of the Phase with ID ‘PhaseID’. Names are still to be standardized. Names are character strings e.g. ‘FCC_A1’, or ‘BCC-A2’.

Information about the fractions of the different phases in the RVE like Crystalline_Fraction, Solid_Fraction can be derived from descriptors depicted in the ensemble section (2.2) according to the scheme described in section 5.

#### 2.1.4. Phase state and crystallinity of the RVE

The most general case of a material is an RVE comprising multiple phases, where each of the phases may comprise multiple features (e.g. solid grains). However, also fluids, gases, single crystals or amorphous states are ‘materials’ which have to be considered. This makes the specification of a PhaseState a meaningful classification.

##### 2.1.4.1. PhaseStateID/PhaseStateName

Refers to the overall phase state in the RVE. Please note that the descriptors given in the present article also would allow describing the geometry of microsystems, e.g. microfluidic devices revealing solid channels for the transport of liquids being in contact with a gas or similar complex settings (Table 3).

**Table 3.** List of PhaseStateIDs and PhaseStateNames providing a rough classification of the material being present in the RVE in terms of its phase state.

PhaseStateName	PhaseStateID	Examples
Not specified	0	
Gas	1	Noble gases, technical gases, molecular gases
Liquid	2	Solutions, melts
Solid	3	Single phase solid materials e.g. solar silicon
MultipleLiquids	4	Emulsions
MultipleSolids	5	Technical alloys, composites
GasLiquidMixtures	6	Vapor, melts with trapped gases
GasSolidMixtures	7	Powders, foams, smoke
LiquidSolidMixtures	8	Dispersions, solidifying melts, mushy zones
Others	9	
Extendable	….	

##### 2.1.4.2. CrystallinityTypeName/CrystallinityTypeID

CrystallinityTypeID and CrystallinityTypeName refer to the overall general crystallographic structure of the solid phases in the RVE.

Descriptors related to the crystal structures of the individual phases like lattice parameters, crystal symmetry, chemical ordering within the crystals, and others are discussed in section 2.2 on ensembles/phases (Table 4).

**Table 4.** CrystallinityTypeIDs and CrystallinityTypeNames for an overall general crystallographic structure of the *solid phases* in the RVE.

CrystallinityTypeName	CrystallinityTypeID	Examples
Not specified	0	
Amorphous	1	Glasses
Semicrystalline	2	Thermoplastics (partially amorphous)
Polycrystalline	3	Polycrystalline solar cells
Polycrystalline textured	4	Rolled products, directionally solidified products
Single crystalline	5	Single crystals
Single crystalline with inclusions	6	Technical single crystals e.g. turbine blades
Multiple twinned	7	Martensite
Complex molecules	8	
Others	9	
To be extended	….	

##### 2.1.4.3. NumberFeatures

Specifies the total number of features in the RVE. A feature is a region of the RVE which can be described with at least one common attribute and thus reveals a contrast to its surrounding with respect to at least one property. This contrast might be the contrast of a physical property like a crystallographic orientation, but it might also just be a name being given to that particular region. An example is a grain of a phase, where the volume/area belonging to that particular grain will have the same identifier (see FeatureID).

#### 2.1.5. Defect structures in the RVE

In discrete models like discrete dislocation dynamics models, ‘defects’ in general are individually resolved as ‘features’ having their own position(centroid), their own orientation (e.g. Burgers vector of a dislocation), a volume and a surface (e.g. of a precipitate), a composition (e.g. of a precipitate) and other attributes (see section 2.3 on FeatureData).

Defects known to be present in the RVE but not being described as spatially resolved ‘features’ can be treated statistically as numbers or by continuous fields like local defect densities. The following descriptors are suggested for this purpose. See the descriptor relations section (section 5) for further description of defects.

##### 2.1.5.1. NumberDefectTypes

Provides the number of different types of defects.

##### 2.1.5.2. DefectTypeID

Identifies the type of defect. The present preliminary specification of different defect types only provides a first basic structure and will need future refinement. Negative values for a specific DefectTypeID indicate defects which may annihilate with their positive counterparts, e.g. DefectTypeID with value 102 (left-handed screw dislocation) can annihilate with DefectTypeID with value –102 (right-handed screw dislocation) (Table 5).

**Table 5.** List of DefectTypeNames and DefectTypeIDs.

DefectTypeName	DefectTypeID	Examples
Point defects	000	Point defects general
	001	
	002	
LineDefects	100	Line defect general
	101	Dislocation
	102	Screw dislocation (left-handed)
	−102	Screw dislocation (right-handed)
PlanarDefects	200	Planar defect general
	…	
Volumetric defects	300	Volumetric defects
	301	Pores
	302	Precipitates

##### 2.1.5.3. NumberDefects(DefectTypeID)

Specifies the number of defects of a given defect type in the RVE.

##### 2.1.5.4. Defect_Density(DefectTypeID)

A derived descriptor that provides the number of defects per volume. Note that for line defects the unit will be length/volume and for planar defects the unit will be area/volume. For point defects and volumetric defects the unit will be 1/volume. At the hierarchical level of the RVE Defect_Density(TypeID) corresponds to the average density of defects of type DefectTypeID in the entire RVE.

#### 2.1.6. Statistics of the RVE

Statistical information about the RVE can be extracted from the information at the lower hierarchical levels, i.e. ensemble, features, and cells. Some relevant descriptor relations like ‘_Density’ or ‘_Fraction’ have already been introduced above. Following the scheme for the derivation of descriptor relations detailed in section 5 a number of relevant entities can be derived.

Descriptor relations like Feature_Size_Distribution would be an array of dimension NumberFeatures with its components being Feature_Size(FeatureID). This concept allows further operations such as averaging relations (e.g. Feature_Size_Average_(FeatureID)) or finding minimum and maximum and many more. Similar operations can be thought of in order to define e.g. orientation distribution functions.

This part of the specification of the descriptor ontology will need much more attention in the future. The concept of a minimum set of basic descriptors allowing derivation of anything of interest seems however to hold.

### 2.2. Ensemble/phase data

**Figure 9.**
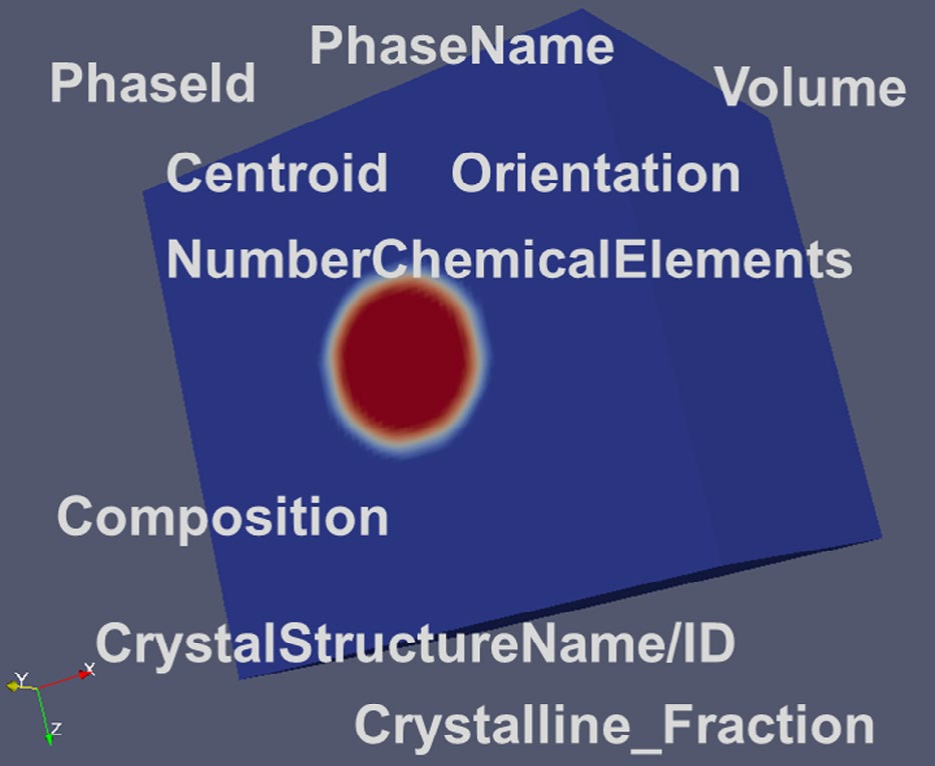
Major descriptors for an ensemble/a phase. The phases (blue and red) are already depicted here in a spatially resolved way.

Ensembles represent the next hierarchical level below the level of the RVE. They have their own data container, which further comprises separate containers for each individual phase. The descriptors are essentially identical with those being defined for the RVE although they may take different values for the ensemble in contrast to the values for the same descriptor at the RVE level. Descriptors being specified for any phase data container are depicted in Figure 9.

#### 2.2.1. NumberConstituents

Indicates the number of constituents in the Ensemble. The NumberConstituents may be less or equal to the NumberConstituents defined for the RVE.

#### 2.2.2. ConstituentID

Specifies the ConstituentID for each constituent in the Ensemble. Runs from 1 to NumberConstituents. NumberConstituents in the Ensemble may be less or equal to NumberConstituents in the RVE, i.e. a subset of the constituents of the RVE. The ConstituentIDs are however the same as used for the RVE.

#### 2.2.3. ConstituentName (ConstituentID)

Specifies the name of the Constituent with ID ‘ConstituentID’. ConstituentNames and ConstituentIDs are the same as those used at the RVE level to ensure that ConstituentNames and IDs are unique and on ensemble level only a subset can be accessed.

#### 2.2.4. PhaseID

The identifier of that particular phase as fixed in the RVE description Each PhaseID is associated with a separate data container comprising all information according to the descriptors listed below.

#### 2.2.5. PhaseName

The name of this particular phase. This name is only repeated here and has to align with the PhaseName(PhaseID) given in the RVE descriptors.

#### 2.2.6. Volume

The volume of this particular phase with PhaseID.

#### 2.2.7. Centroid

A vector describing the geometric center of this phase in the RVE Frame. Perfect emulsions of two liquid phases would have the same centroid, whereas separated phases would reveal different centroids.

#### 2.2.8. Orientation(OrientationTypeID) or Orientation(OrientationTypeName)

Provides the overall orientation of this phase. Makes sense in case the phase reveals an anisotropy leading to a preferred direction. Such a preferred direction may be caused by a topological anisotropy of the features of that phase, e.g. a fiber type arrangement, and/or by a crystallographic anisotropy, such as a rolling texture.

#### 2.2.9. NumberChemicalElements

The number of chemical elements present in this phase. This number may be less or equal to the NumberChemicalElements specified for the RVE. However, CEID and ChemicalElementName(CEID) are the same as used in the RVE.

#### 2.2.10. AtomPercent(CEID)

Provides the relative abundance of a chemical element with CEID in this phase in atom %.

#### 2.2.11. Composition(unit)

Composition(AtomPercent) and Composition(MassPercent) are vectors describing the relative abundance of the different chemical elements in a given system, which is the particular phase at the ensemble level. They are specified via a ‘unit’ attribute (see section 5.3) in either AtomPercent (unit=at.%) or in MassPercent (unit=wt.%), respectively. The dimension of these vectors corresponds to the NumberChemicalElements.

#### 2.2.12. CrystalStructureName/CrystalStructureID

CrystalStructureName and CrystalStructureID specify the crystal structure and hold e.g. for solids (PhaseStateID=3) which are either polycrystalline (CrystallinityTypeID=3) or single crystalline (CrystallinityTypeID = 5).

Respective IDs by now are only specified for simple and frequent crystallographic structures such as fcc, bcc, and hcp. Complex crystallographic structures can be defined by suitable and standardized schemes like Crystallographic Information Files (CIF) [24, 25] or .xyz files as used in RasMol or JMol (Table 6).[26,27]

**Table 6.** CrystalStructureNames and CrystalStructureIDs.

CrystalStructureName	CrystalStructureID	Examples
Not specified	0	
FaceCenteredCubic fcc	1	Austenite, Ti(C,N)
BodyCenteredCubic bcc	2	Ferrite
HexagonalClosedPack hcp	3	Magnesium crystals
Orthorhombic	4	
…	..	
Complex specification	9	Use of crystallographic information files [24, 25]
To be extended	….	

#### 2.2.13. ChemicalOrderingName/ChemicalOrderingID

ChemicalOrderingName classifies the type of ordering of the chemical elements within the crystal structure of a specific phase (Table 7).

**Table 7.** ChemicalOrderingName and ChemicalOrderingIDs.

ChemicalOrderingName	ChemicalOrderingID	Examples
Not specified	0	
Solid solution crystal	1	
Intermetallic compound (fully stoichiometric)	2	Fe_3_C
Intermetallic compound (stoichiometric with respect to one chemical element)	3	Ti(C,N)
...	..	
…	..	
Complex specification	9	
To be extended	….	

#### 2.2.14. CrystalSymmetryName/CrystalSymmetryID

CrystalSymmetryName classifies the symmetry of the crystal structure and is by limited to simple cases with options to extensions (Table 8).

**Table 8.** CrystalSymmetryName and CrystalSymmetryIDs.

CrystalSymmetryName	CrystalSymmetryID	Examples
Not specified	0	
Cubic	1	
Hexagonal	2	
Orthorhombic	3	
...	..	
…	..	
…	..	
…	..	
…	..	
Complex specification	9	
To be extended	….	

#### 2.2.15. Crystalline_Fraction

Defines the fraction of crystallized volume with respect to the overall volume of this ensemble respectively phase. This descriptor finds applications especially in thermoplastics.

#### 2.2.16. LatticeConstants(PhaseID)

A simple three-component vector specifying the lattice constants of this particular phase. This descriptor and its values are meant to be applied to simple crystal structures only. Complex crystal structures will need more detailed descriptions, like CIF data files [24,25] or ‘.xyz’ files.[26,27]

### 2.3. Feature data

**Figure 10.**
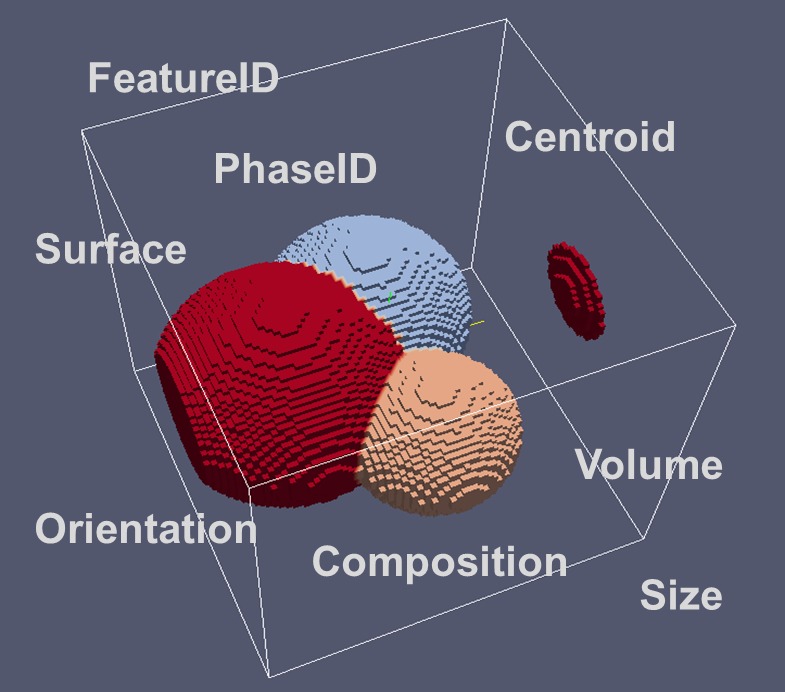
Descriptors for Features in an RVE. Note the similarity with the descriptors for the RVE geometry.

Each phase in nature or in laboratory experiments may be present as a single crystal allowing derivation of the properties of this phase. These per-phase properties have been collected in the preceding ensembles section. In general materials, however, are polycrystalline and reveal numerous grains of the same phase, which can be observed under a microscope. These grains represent ‘features’ of the microstructure (Figure 10). Other features can be defects, e.g. pores. At very high resolution also very small defects like dislocations or even individual atoms can be treated as features in principle.

Most of the descriptors being specified for the individual features are identical to those descriptors being specified for the RVE and the ensemble/phases. They will just take different values, e.g. the composition of an individual grain may and will differ from the average composition of all grains of a phase – the composition of the ensemble. The composition of the RVE in turn will be the average composition of all phases in the RVE weighted by their volume fractions.

Some descriptors are required to account for the presence or absence of a feature in a particular region of the RVE and accordingly for a spatially resolved description.

A feature here is defined as a coherent region of space, which can be distinguished from other regions due to a contrast in at least one attribute/property. The feature further has at least one attribute/property which is identical for each point of space occupied by the feature. This attribute may be the ‘name’ of the feature indicating that somebody – based on some criterion – has already identified the region to belong to the same physical object.

#### 2.3.1. Feature_Size

Defines the size of a feature as the radius of an equivalent sphere revealing the same volume. This descriptor is thus a derived descriptor and is very useful for statistical purposes, e.g. the calculation of Size_Distributions. This descriptor may further profit from additional attributes like ‘MinimumValue’ and ‘MaximumValue’ which allow introducing anisotropy. See section 6.1 on descriptor attributes.

#### 2.3.2. FeatureID

Denotes the unique identifier for this particular feature. In regions of the RVE where this feature is present, the feature indicator function (see section on Field Data) takes the value FeatureID. Negative values for the FeatureID correspond to *features outside the RVE.* The value 0 may not be used for the feature ID at the present Feature Data level.

#### 2.3.3. Volume

The volume of this feature.

#### 2.3.4. Centroid

The geometric center of this feature in the RVE Frame*.*

#### 2.3.5. Orientation(OrientationTypeID) or Orientation(OrientationTypeName)

The average orientation of a defined lattice vector (e.g. [100]) or direction of anisotropy of this feature with respect to the orientation of the RVE.

#### 2.3.6. AtomPercent(CEID)

Relative abundance of a chemical element with CEID in this feature.

#### 2.3.7. Composition(unit)

Composition(unit=at.%) and Composition(unit=wt.%) are vectors describing the relative abundance of the different chemical elements in a given system, which is the feature in the present case.

### 2.4. Field data

#### 2.4.1. Discretization of the RVE

In general, NumericalElements are the smallest volume element being described in the dataset. These NumericalElements are used to build up features, ensembles and eventually the entire RVE. The RVE may have arbitrary shape and also all the NumericalElements used for its discretization (e.g. Finite Elements and Voxels) may have arbitrary shape. The NumericalElements not necessarily have to be voxels and they may be defined by vortices, edges and faces [see e.g. 9, 10]. Descriptors for fields thus can be specified *independent of any discretization scheme*.

**Figure 11.**
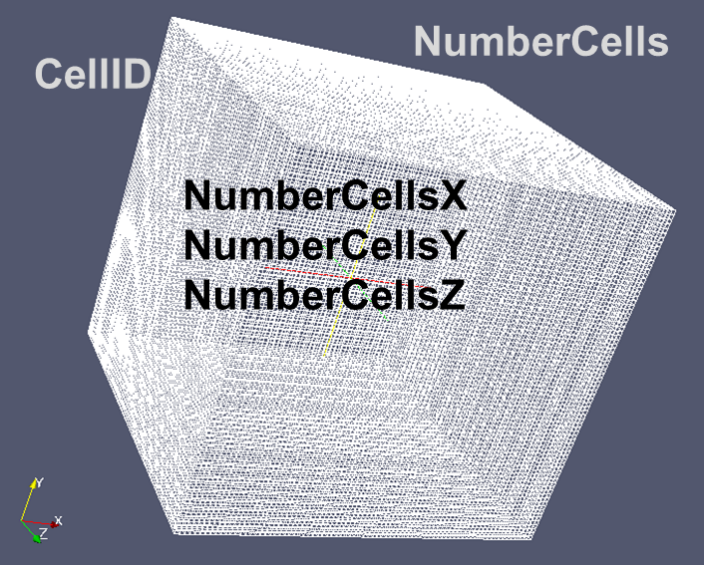
Descriptors for a discretized simple geometry.

Numerous experimental data and also microstructure simulations are available in a voxel representation (Volume pixels, i.e. small cubes). The intuitive understandability of such a voxel scheme makes this representation well suited for interdisciplinary discussions with scientists and engineers not well skilled in numerical methods. All descriptors depicted in the present paper have been derived, discussed, defined and interpreted having a simple numerical representation in regular structured grids composed of Voxels called ‘Cells’ in mind for volume type data descriptors (Figure 11).

##### 2.4.1.1. NumericalElementID

NumericalElementID (NEID) specifies the ID of a specific NumericalElement in the RVE. Runs from 1 to NumberNumericalElements.

Or, alternatively, for Voxel based representations:

##### 2.4.1.2. CellID

Special type of NEID for voxel type representations: specifies the ID of a specific cell (Voxel) in the RVE. Runs from 1 to NumberCells.

##### 2.4.1.3. NumberNumericalElements or NumberCells

Specifies the total number of volumetric numerical elements respectively cells describing the RVE.

##### 2.4.1.4. NumberCellsX, NumberCellsY, NumberCellsZ

Only for a simple rectangular voxel type grid NumberCells=NumberCellsX*NumberCellsY*NumberCellsZ

##### 2.4.1.5. CellSize

Defines the size/scaling (e.g. micrometer/cell) of a single numerical cell in the RVE for a voxel type grid. To be specified in near future for geometries discretized by isoparametric finite elements.

##### 2.4.1.6. CellSizeX, CellSizeY, CellSizeZ

Used only for simple geometries (Voxels): CellSize=CellSizeX=CellSizeY=CellSizeZ given in e.g. micrometer/cell or as specified by attributes (see section 5.2).

#### 2.4.2. Describing continuum fields

Once a simple – or even complex – discretized geometry of the RVE is available, values can be assigned to the individual CellIDs for scalar, vector and tensor fields varying continuously in space.

A central role as a continuum field takes the FeatureID field as it identifies all cells in the discretized space belonging to the same feature. It thus provides the option to describe a number of discrete objects by a single continuum function.

##### 2.4.2.1. FeatureID

**Figure 12.**
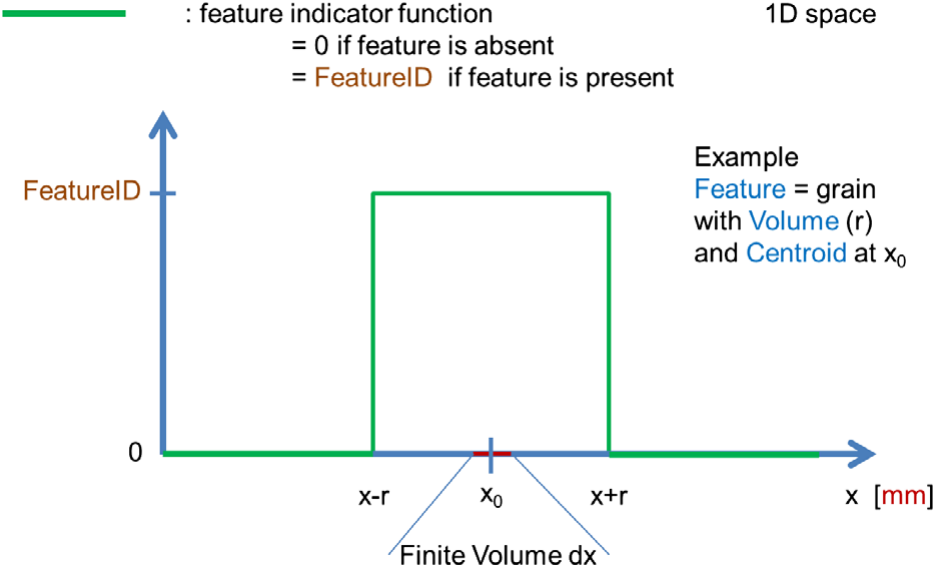
FeatureID: Graphical scheme of a Feature indicator function in a 1D representation. The finite volume corresponds to a NumericalElement.

The FeatureID function is a continuous function in real space. It takes the value FeatureID if a Feature with FeatureID is present and 0 else. The transition between the values ‘0’ and ‘FeatureID’ not necessarily has to be a step function but also can be continuous function (Figure 12).

##### 2.4.2.2. FeatureID_Fraction(FeatureID)

The derived descriptor FeatureID_Fraction, i.e. FeatureID(Field)/FeatureID(FeatureData) corresponds to a continuous field describing the fraction of a feature and takes values between 0 and 1. This corresponds to approaches used in e.g. phase-field models.[28]

##### 2.4.2.3. Orientation(OrientationTypeID) or Orientation(OrientationTypeName)

Describes the local orientation at each point in space. This field is relevant e.g. for twinned structures and for subgrains where the orientation varies within the feature.

##### 2.4.2.4. AtomPercent(CEID)

Describes the local relative abundance of the chemical element with CEID in each cell in atom %. This field is used especially for the description of diffusion processes.

##### 2.4.2.5. LatticeParameters

Describes the local values of the LatticeParameters in each cell. This field is relevant e.g. for stresses/strains, for thermal expansion, for diffusion and many others. Typically this value will not be used, but the descriptor ‘strain’ is used instead.

##### 2.4.2.6. Strain and StrainTensor

A derived descriptor that describes the local deviation from the equilibrium LatticeParameters, i.e.:

$$\text{Strain}=100^{*}\text{(LatticeParameters(NumericalElement)}-\text{LatticeParameters(Ensemble))}/\text{LatticeParameters(Ensemble))}$$

This strain definition is only valid when a small strain formulation is adopted. More general is the specification of a full StrainTensor, which allows shear deformations also to be considered.

##### 2.4.2.7. Defect_Density(type)

Provides the local density of defects of the given type in the cell volume. See further specification of this descriptor in the section on the RVE level.

##### 2.4.2.8. FlowField

Describes the actual (for the given instant) local velocity vector of the flow for fluids in each cell.

In the future numerous other fields will have to be specified beyond the mere geometric data collected in the present article. Examples for such fields and possible descriptors could read: TemperatureField, ElectricField, MagneticField, StressField. Also any property of a phase as defined by a future property descriptor list may vary in space and then can be represented by a respective field.

## 3. Surface and interface data

Surfaces and interfaces between different three dimensional features play an important role for the properties of polycrystalline and multiphase materials. Frequently, properties and fields vary discontinuously across such boundaries. Examples are the heat transfer coefficient, the electrical contact resistivity or mechanical stresses. Interfaces also play an important role for the evolution of microstructures in terms of minimizing interfacial areas. Examples are the structure of soap bubbles in a foam or coarsening of grain structures in metals and alloys.

**Figure 13.**
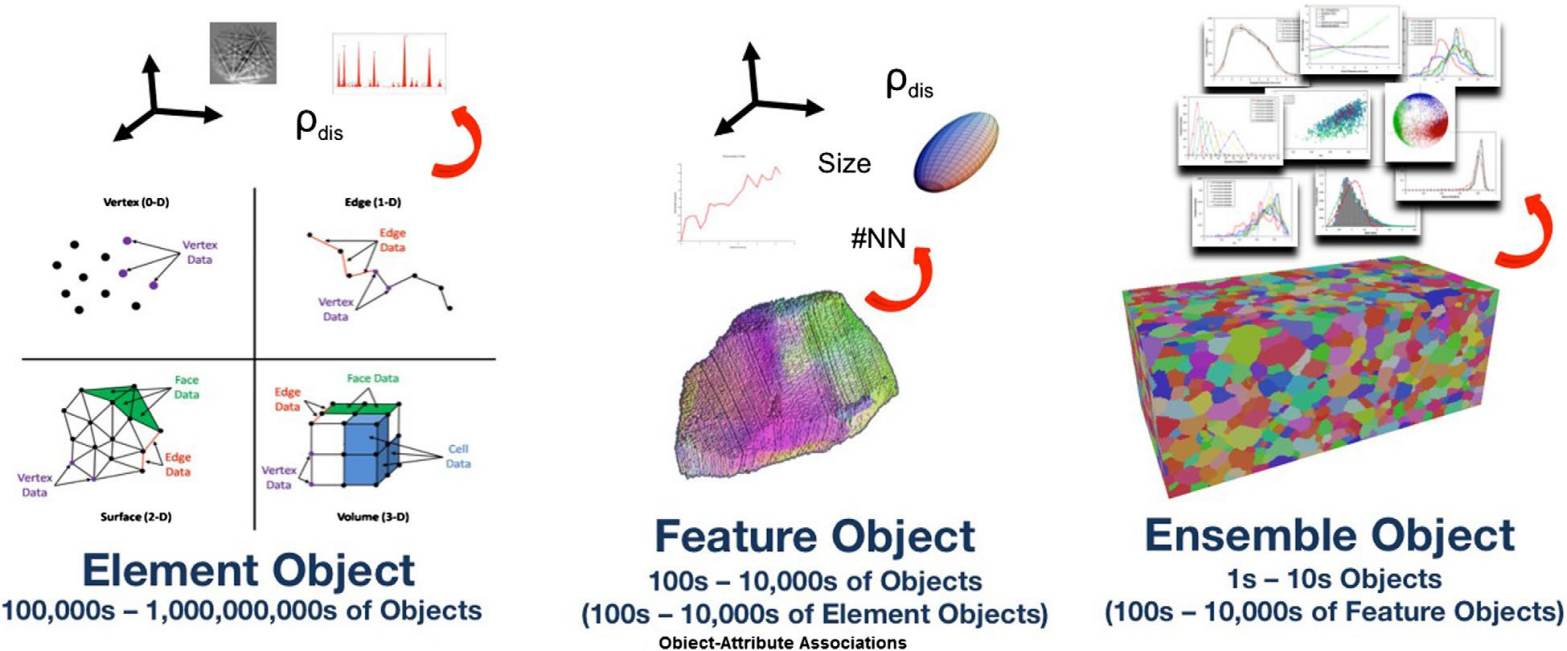
Construction of volumetric NumericalElements (Cells) from vortices, edges, and surfaces (left: element object), further assembly of a number of NumericalElements forming a Feature (middle: feature object), and eventually an Ensemble of Features filling the entire RVE in this case.[10]

Thus there is a strong need to specify descriptors for a spatially resolved description of 2D (surfaces/interfaces; sections 3.1–3.3), 1D (lines/edges; section 3.4) and 0D (points/vortices; section 3.5) structures in a similar hierarchical manner as described for the 3D data in section 2. For the numerical representation a respective approach has been made in Dream3D [9, 10] (Figure 13).

Interfacial properties such as ‘InterfacialEnergy’ or ‘HeatTransferCoefficient’ can then later be assigned to each of the respective descriptors once a basic list of such property descriptors is available.

### 3.1. Faces – the 2D NumericalElements

The smallest 2D element is called a Face. Similar to the NumericalElements or Cells in the 3D situation depicted in section 2, Faces correspond to the highest resolution for the description of 2D structures in the given hierarchy. A number of descriptors defined for the 3D description are found again for 2D descriptors.

**Figure 14.**
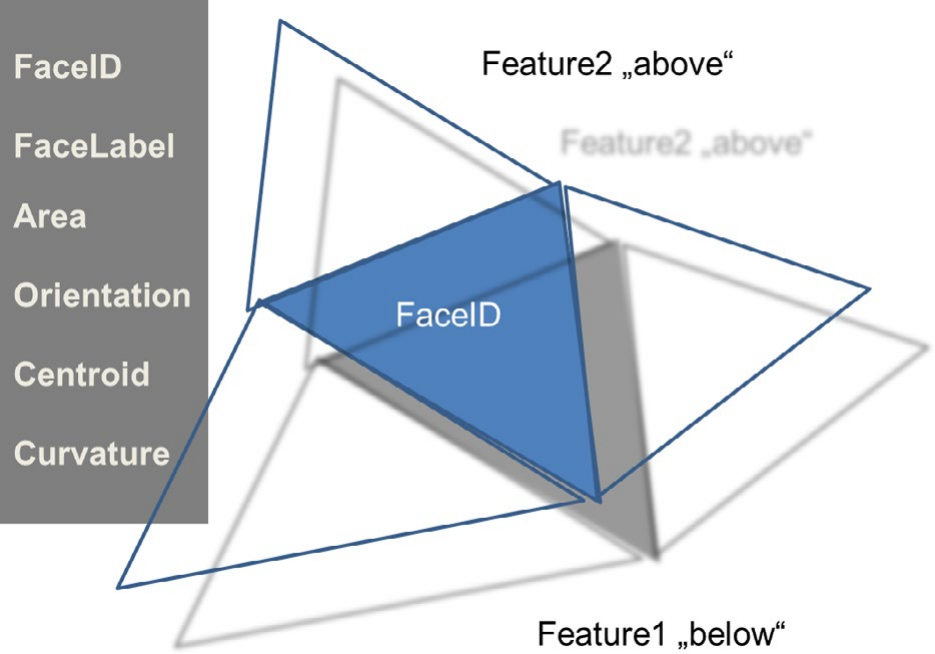
Some major descriptors for Faces.

Descriptors related to surfaces and interfaces are discussed having one or more unstructured grids of points in mind, forming ‘triangles’ as smallest surface elements which are denoted as ‘Faces’ in a general formulation. Although all subsequent discussions are based on this ‘triangle’ the descriptors being defined are generic and independent of any specific digital representation (Figure 14).

#### 3.1.1. FaceID

A unique identifier for the individual surface element respectively for each Face. This ID originates from the discretization scheme.

#### 3.1.2. FaceLabel

Each Face is associated with a FaceLabel specifying which two half-spaces respectively Features the Face separates. The ‘FeatureIDs’ are identical to the FeatureIDs specified for the bulk 3D features. The sequence of the FeatureIDs has a meaning with respect to the Orientation of the FaceNormal. For FaceLabel(Feature1,Feature2) the FaceNormal points from Feature1 to Feature2.

#### 3.1.3. Area

Denotes the area of this particular Face.

#### 3.1.4. Centroid

Specifies the location of the centroid of the Face. The centroid will be in the same plane for planar Faces.

#### 3.1.5. NormalVector

Denotes the Normal vector of the Face. The direction of this vector is specified by the FaceLabel descriptor. Note that the orientation of surface elements in numerical discretization schemes often depends on the winding scheme of the underlying nodes/vortices.

#### 3.1.6. Curvature

This descriptor provides the options to add further information in the case of non-planar Faces. Similar to ‘FaceNormal’, ‘FaceLabel’ defines the sign/type of curvature. Convex curvature – as seen in the direction from Feature1 to Feature2 – is assigned with a positive value. The same Face seen in the direction from Feature2 to Feature1 would be concave and have a negative curvature value. More complex descriptions of the FaceCurvature, e.g. minimum and maximum curvature or shape functions, can be assigned in future to this descriptor using additional attributes (see section 5.3).

#### 3.1.7. Orientation(OrientationTypeID) or Orientation(OrientationTypeName)

Provides the orientation of the FaceNormal in the RVE ReferenceFrame.

#### 3.1.8. Thickness

This descriptor has been introduced to allow for the handling of deviations from ideal two dimensional behavior of the interface description being depicted in this section. The meaning of thickness may range from the thickness of a diffuse interface in phase-field models up to the surface roughness of technical materials. More work is needed here in future.

### 3.2. FaceFeatures – the 2D Features

FaceFeatures are the 2D counterparts of the 3D Features. Similar to the definition of the Features, which are defined as regions in the RVE having at least one common characteristic, the FaceFeatures are a set of Faces describing a 2D area having a common characteristic. Examples are a grain boundary between two grains or a part of the RVE boundary (e.g. one face of the simple RVE cube).

#### 3.2.1. FaceFeatureLabel (Feature1ID, Feature2ID)

Defines all Faces belonging to the interface area between Feature1 and Feature2. For example, FaceFeatureLabel(FeatureID1,FeatureID2) corresponds to a grain boundary between Feature1 (grain 1) and Feature2 (grain 2) in the case of two grains belonging to the same phase. The orientations of the two individual features allow the determination of the relative misorientation between the grains and thus the specification of the type of grain boundary.

#### 3.2.2. Area (FaceFeatureLabel)

Specifies the interface area between FeatureID1 and FeatureID2.

#### 3.2.3. InterfaceType

A vector comprising the three integer components denoted as InterfaceType1ID, InterfaceType2ID and InterfaceType3ID. It specifies interface dimensionality and different types of interfaces and surfaces. Table 9 provides a preliminary categorization which needs to be further elaborated in the future.

**Table 9.** Interface types and their IDs.

InterfaceType	InterfaceType1ID (dimensionality)	InterfaceType2ID	InterfaceType3ID
2D interfaces			
No interfaces/not specified	Any	0	0
Grain boundary	2	1	0
Low angle GB	2		1
High angle GB	2		2
Coincident site lattice (CSL) Boundary (general)	2		3
Sigma 3 boundary	2		4
Sigma 7 boundary	2		5
Epitaxial layers	2		9
Phase boundary	2	2	0
Coherent	2		1
Incoherent	2		2
Epitaxial layers	2		9
RVE surface	2	7	
1D interfaces			
Triple line	1		To be defined
RVE edge	1		To be defined
0D interfaces			
Quadruple point	0		To be defined
RVE corner	0		To be defined
All interfaces	99	99	99

All other descriptors specified for the Faces e.g. NormalVector, Orientation, Curvature can be used also for FaceFeatures wherever this seems meaningful (Table 9).

### 3.3. Interfaces and surfaces – the 2D ensembles

The next higher level in the hierarchical description of 2D structures are ensembles allowing descriptions of surfaces and interfaces being independent of the individual 3D features they are separating. Examples are all interfaces between two 3D ensembles/phases or the total surface of an individual feature.

#### 3.3.1. Interface(PhaseID1,PhaseID2)

Describes the total interface between two different phases in the RVE. Can be defined via the Faces or via the FaceFeatureLabels as:

All Faces – or FaceFeatureLabels – for all pairs of FeatureIDs where FeatureID1 belongs to PhaseID1 and FeatureID2 belongs to PhaseID2 (and FeatureID1 belongs to PhaseID2 and FeatureID2 belongs to PhaseID1).

#### 3.3.2. Surface (FeatureID)

**Figure 15.**
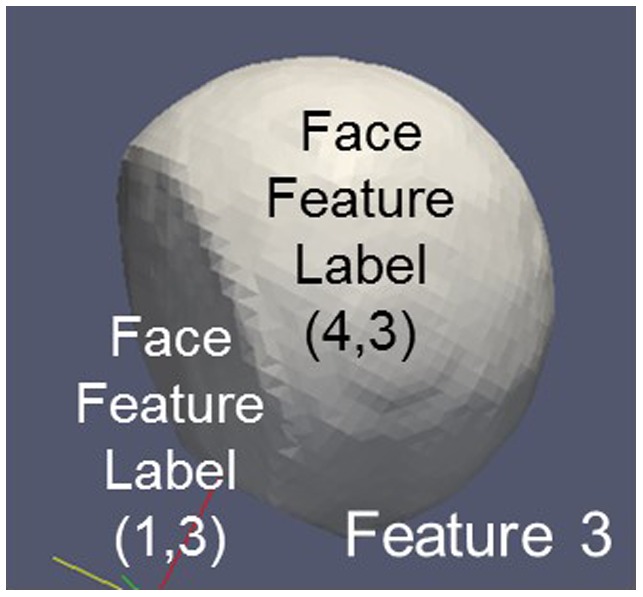
Surface of a Feature 3 being composed from different interface areas identified by different FaceFeatureLabels. Feature 4 (liquid) is not shown. FaceFeatureLabels to be combined are FaceFeatureLabel(3,*) and FaceFeatureLabel(*,3) where * denotes all FeatureIDs except 3.

Specifies the total surface of a Feature, which in general will be composed of different FaceFeatures having their individual FaceFeatureLabels (Figure 15). Negative values for the FeatureID denoting Features outside the RVE or RVE boundaries have to be handled with care. Total surfaces of a feature are helpful to assess fluxes from/into the feature or to estimate deviations from spherical behavior.

#### 3.3.3. SurfaceArea (FeatureID)

Specifies the total surface area of the feature with FeatureID.

#### 3.3.4. InterfaceArea (PhaseID1,PhaseID2)

Specifies the interface area between the phases with PhaseID1 and PhaseID2.

All other descriptors specified for the Faces, e.g. NormalVector, Orientation, and Curvature, can be used also for above Surfaces and Interfaces wherever this seems meaningful.

### 3.4. Triple junctions

The following section goes further down in the dimensional hierarchical description of any material. Subsequent to the 3D and 2D data structures depicted in the previous sections it introduces 1D line-type and 0D point-type like features. Please note that there is a priori no relation to the 0D vortices and 1D edges entering into the numerical discretization, e.g. in Dream-3D (see also Figure 13).

**Figure 16.**
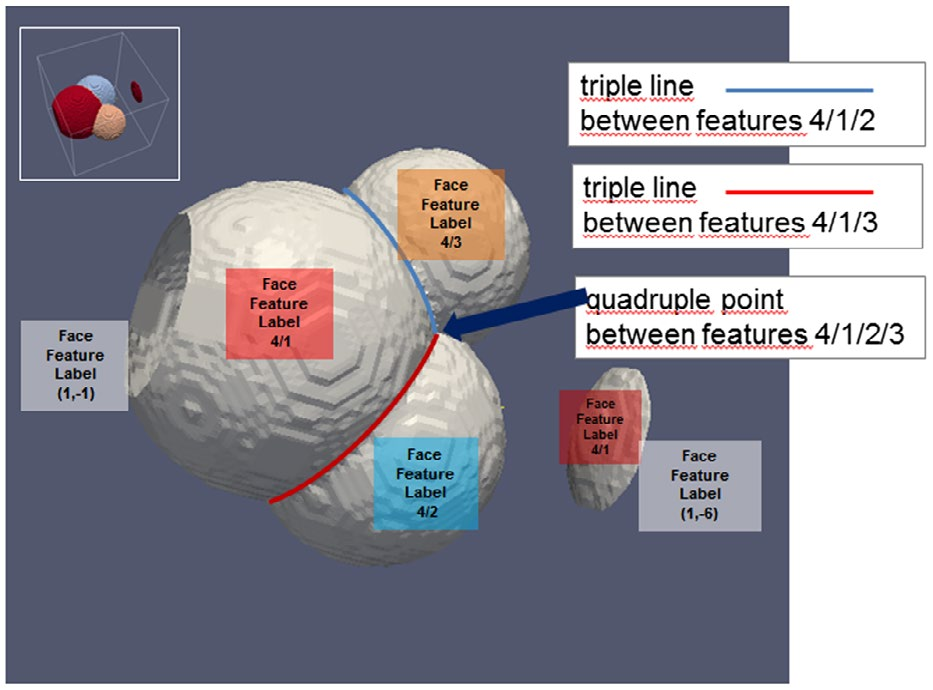
FaceFeatureLabels for the different Features of the example. Feature 4 (Liquid Phase) is not shown. Note the negative FeatureIDs representing Features outside the RVE boundaries.

Triple lines occur in case of three features coexisting in 3D space and QuadruplePoints appear at the coexistence of four features (see also Figure 16). TripleLines and QuadruplePoints are characteristic features of most microstructures and exhibit, for example, special conditions for nucleation of new phases, making their individual description valuable.

#### 3.4.1. TripleLineSegmentID

Defines an identifier for each TripleLineSegment

#### 3.4.2. TripleLineSegmentLabel(FeatureID1,FeatureID2,FeatureID3)

Denotes a short – in general linear and straight – segment of a complex triple line separating the three features.

#### 3.4.3. Centroid

Denotes the position of the Centroid of the TripleLineSegment.

#### 3.4.4. Length

Provides the length of the TripleLineSegment.

#### 3.4.5. Orientation(OrientationTypeID) or Orientation(OrientationTypeName)

Defines the Orientation of the TripleLineSegment orientation vector with respect to the RVE ReferenceFrame. The sign of the orientation vector must be specified if needed.

#### 3.4.6. Curvature

Optionally describes the curvature of the TripleLineSegment. Needs further elaboration if needed.

#### 3.4.7. TripleLineLength(FeatureID1,FeatureID2,FeatureID3)

Defines the total length of the triple line as a sum of the lengths of all its TripleLineSegments.

### 3.5. Quadruple junctions

#### 3.5.1. QuadruplePointID

Defines an identifier for each QuadruplePoint.

#### 3.5.2. Centroid

Denotes the position of the QuadruplePoint

### 3.6. RVE boundaries and interface statistics

The 2D equivalents of the descriptors describing the 3D RVE are descriptors describing surfaces and interfaces related to the entire RVE. Examples are the RVE boundaries and statistics on interfaces, triple lines and quadruple points.

#### 3.6.1. Surface

Describes the entire surface of the RVE to which boundary conditions can be applied. RVE boundaries correspond to all Faces(FeatureId1,FeatureID2) respectively FaceFeatureLabels (FeatureId1, FeatureID2) where at least one of the FeatureIDs has a negative value. Note that FeatureIDs of features located outside the RVE are negative.

#### 3.6.2. SurfaceArea

Defines the total surface area of the RVE. For simple rectangular geometries in a voxel type representation the surface area can be calculated as:

$$\text{SurfaceArea}=2^{*}{\text{NumberCellsX}}^{}*{\text{CellSizeX}}^{*}{\text{NumberCellsY}}^{*}\text{CellSizeY}+2^{*}{\text{NumberCellsZ}}^{*}{\text{CellSizeZ}}^{*}{\text{NumberCellsX}}^{*}\text{CellSizeX}+2^{*}{\text{NumberCellsY}}^{*}{\text{CellSizeY}}^{*}{\text{NumberCellsZ}}^{*}\text{CellSizeZ}$$

#### 3.6.3. NumberSurfaces

Specifies the number of different surface ensembles forming the boundary of the RVE. Would equal six for a simple cubic RVE, e.g. SurfaceNorth/South, SurfaceEast/WestY, SurfaceTop/Bottom

#### 3.6.4. SurfaceArea(FaceFeatureLabel)

Specifies the part of the surface area of the RVE being defined by the ensemble FaceFeatureLabel.

#### 3.6.5. InterfaceArea

Defines the total area of all interfaces of all InterFaceTypes being present in the RVE. This is an interesting entity to define an InterfaceArea_Density. See section on derived descriptors.

#### 3.6.6. NumberInterfacesTypes

Specifies the total number of different InterfaceTypes present in the RVE, e.g. all grain boundaries and phase boundaries forming the 2D ensembles.

#### 3.6.7. InterfaceArea(InterfaceType)

Defines the total area of all interfaces of a given InterfaceType present in the RVE.

#### 3.6.8. SurfaceNormal(FaceFeatureLabel)

Specifies the Normal vector of a surface defined by FaceFeatureLabel. Only meaningful if the Surface defined by FaceFeatureLabel can be considered as planar.

#### 3.6.9. Edges

Specifies the edges of the RVE as line segments (vectors). Currently meant for simple geometries only. To be extended to more complex RVE geometries in future.

#### 3.6.10. Corners

Specifies the corners of the RVE. Currently meant for simple geometries only. To be extended to more complex RVE geometries in future.

#### 3.6.11. BoundaryConditionType

Another relevant topic is the definition of boundary conditions on the RVE level. BoundaryConditionType is a vector comprising the two integer components denoted as BoundaryConditionType1ID and BoundaryConditionType2ID. It specifies the type of boundary conditions applied to the RVE surfaces. Table 10 provides a preliminary categorization which should be further elaborated in the future (Table 10).

**Table 10.** BoundaryConditionTypes and BoundaryConditionTypeIDs.

BoundaryConditionType	BoundaryCondition Type 1ID	BoundaryCondition Type 2ID
Periodic	1	To be defined
Symmetric	2	To be defined
Flux	3	To be defined
Gradient	4	To be defined
Fix	5	To be defined
Uniform extension	6	To be defined
Surface traction	7	To be defined
Other	99	

## 4. Minimum set of basic descriptors

The present article has identified a number of descriptors which in the following are cast into a minimum set of descriptors being sufficient to comprehensively describe the geometric structure of a static material. The descriptors may be used on any of the hierarchical levels of the materials description, i.e. RVE, Ensembles/phases, Features/grains and Fields. A full availability of data for a specific descriptor at the Field-level allows the determination of the data for this particular descriptor at all higher level. The following summarizes the most important descriptors specified in the present article giving the relations between the *fundamental descriptors* being based on mere numbers, IDs and others, the *scientific descriptors* drawing on some derived descriptors (see section 5) in view of applications in science and the *engineering descriptors* introducing even a *property* not related to the mere geometry – the mass.

Most of the basic descriptors are understandable and even identical for these three areas, e.g. Centroid, Surface, Volume, Edge, Corner, Orientation, Volume, Area, Length, and the various Names and IDs (Table 11).

**Table 11.** Comparison of the use of descriptors in different representations in different communities of science and engineering.

Descriptor (scientific, discrete atomistic)	Descriptor (scientific, continuum)	Descriptor (engineering)
NumberAtoms	NumberAtoms (in moles)	Mass
AtomPercent	AtomPercent	MassPercent
Composition (AtomPercent)	Composition (AtomPercent)	Composition (MassPercent)
n/a	Curvature	Curvature
n/a	MaterialType	MaterialType
NumberDefects and NumberDefectTypes	Defect_Density(DefectTypeID)	Defect_Density(DefectTypeID)
	NumberDefectTypes	NumberDefectTypes

Major differences occur for descriptors which represent countable entities in discrete atomistic models, such as the NumberAtoms (with unit=1). For continuum models this number becomes huge and in general is given in moles (i.e. unit=moles). The individual atoms become unimportant and only their relative amount is specified using the AtomPercent descriptor. This descriptor can however also be specified for the atomistic models counting individual atoms, thus providing a communication channel between these two different model worlds. The step from a scientific specification of a composition in atom_% toward an engineering type specification of composition in mass_% is made by converting the AtomPercent into a MassPercent entity and by replacing the NumberAtoms (in moles) by the Mass of the system. Any combination of these different descriptors in the different worlds can be used to describe the composition of the system at any scale. Similar arguments hold for a number of defects (e.g. in discrete models) and defect densities in continuum models. Some entities become meaningful only for continuum models while others probably apply only to discrete models.

## 5. Relations between the basic sets of descriptors

The basic set of descriptors, which is meant to be sufficient to describe any microstructure, has been qualified in the preceding sections 2–4. This set forms the basis for an ontology of the field which has to be further complemented by relations between the individual descriptors. A first procedure to generate relations between the different descriptors exploits the benefits of the hierarchical description as depicted in the present paper with the same descriptor being used at different levels of the hierarchy. This allows a relation to be established describing fractions. Please note that there is however *no need to perform these operations* as their values can all be derived when the basic set of descriptors is completely filled with the respective data.

The relations between the different basic descriptors, however, become important if not all the data for the description of the microstructure *by the basic set of descriptors* are available. An example is the composition of a system, which – in typical engineering applications – is given in mass percent of the individual chemical elements (with identifier CEID). This implies the need for creating relations between such engineering values and the values of the basic descriptors, which are the NumberAtoms(CEID) and NumberChemicalElements in this particular case.

The relation for a mass percent composition specification reads:

Mass Percent(CEID)=100×NumberAtoms(CEID)×Mass(CEID)

which is the mass of all atoms of element CEID *divided by the total mass of the system*, which is defined as the sum of the masses for all atoms of all chemical elements: i.e. Sum[NumberAtoms(CEID) × Mass(CEID)] with CEID running from 1 NumberChemicalElements.

Some further examples of operations to generate relations between descriptors for system size invariant entities and simple mathematical operations as specified in the following subsections.

### 5.1. Descriptor relations for size invariant entities

System size invariant entities are very important to transfer data between the different hierarchical levels of the system. Examples for system size invariant entities include fractions, densities, and composition. For any homogeneous, isotropic system these would take distinct values independent of the size of the system. NumberAtoms, in contrast, would increase with increasing system size.

A *fraction relation* is the value of a descriptor at a given hierarchical level being divided by the value of the same descriptor at a higher level of the hierarchy. An example is the volume fraction of a phase in the RVE, where the Volume(PhaseID) is divided by the Volume(RVE). This new entity defined by the relation is important in engineering applications and could be named Volume(PhaseID)_Fraction.

A *density relation* is obtained by normalizing the basic descriptors by volume. An example is a NumberFeature_Density relation, which can be obtained by dividing the NumberFeatures by the Volume thus yielding the number of grains per volume. Defect_Density(DefectTypeID) as another relation provides the density of a specific defect type.

### 5.2. Mathematical operations on descriptors

Simple mathematical operations can provide a number of further useful relations.

A *size relation* calculates/denotes the equivalent size (linear extension) of a feature, an RVE, or an ensemble as the radius of sphere having the same volume. An example is Size(FeatureID)_size=3/(4π)* Volume(FeatureID)_Root3, where root3 denotes the cubic root of the value of the descriptor. In a similar manner numerous further relations of the basic set of descriptors can be defined by simple mathematical operations, such as _root2 providing the square root of the value of the descriptor. Further relations include: _sum, _diff, _product, and _ratio providing the sum, difference, product and ratio of the values of descriptors, respectively. The difference Centroid(Feature1) – Centroid(Feature2) would yield the distance between these two features.

### 5.3. Descriptor attributes

Beside operations acting on the descriptors, a number of further *attributes can be assigned to any of the descriptors* being depicted in this article. The basic scheme for this reads:

Descriptor = Descriptor (attribute1, attribute2, attribute3, …, attributeN).

Such attributes and their values can be easily included into a metadata schema based on the descriptors being defined in the present article once also these attributes and their descriptors are defined in full detail. There is no need to obey a specific sequence for the attributes. In contrast to specifying a descriptor or a sequence of descriptor extensions for each additional detail, e.g. CEID=1; ChemicalElementName(CEID) = Fe; Composition(CEID) = 0.80; CompositionUnit(CEID) = wt.%; CompositionType(CEID)= real, …, metadata schema [29] are easily extendable and amendable to a host of attributes and simultaneously provide both data integrity and data curability.

A metadata scheme for a specific example for attributes of the descriptor ‘composition’ (with values of the attributes indicated by the ‘=’ sign) could read:

Composition (unit = wt.%, Type=Real, NumberChemicalElements=2, ChemicalElementName=Fe, CEID=1, scaling=1, lower bound=0, upper bound=100, error_percent=5, parent=RVE, data origin = experimental, …) = 0.80

One of the descriptors for attributes has already been introduced in sections 2–4 as the attribute descriptor ‘unit’, which for the descriptor Composition may take the values ‘at.%’ or ‘wt.%’. For the descriptor NumberAtoms the descriptor ‘unit’ may take values of ‘1’ or ‘moles’.

### 5.4. Establishing the descriptors – an HDF5 template file

One of the major challenges is to establish a best practice of globally agreed communication and information exchange. For this purpose a metadata scheme as communicated by the present article has to be disseminated widely, but especially there should be no or at least only a low threshold to apply it in practice. Based on the metadata descriptors depicted in this article, which will become part of more comprehensive metadata schemata, the authors thus constructed a preliminary HDF5-file structure as a template based on the simple example of a microstructure of a binary two phase Al-Cu alloy.

As stated in the introduction, an HDF5 type data structure [12] has been identified as a pragmatic approach for a standardized, file based information exchange [13] and the missing link towards a seamless exchange of microstructure information has been closed on the basis of the present article by specifying a unified set of metadata descriptors allowing naming of the different entities in an HDF5 file describing a microstructure.

**Figure 17.**
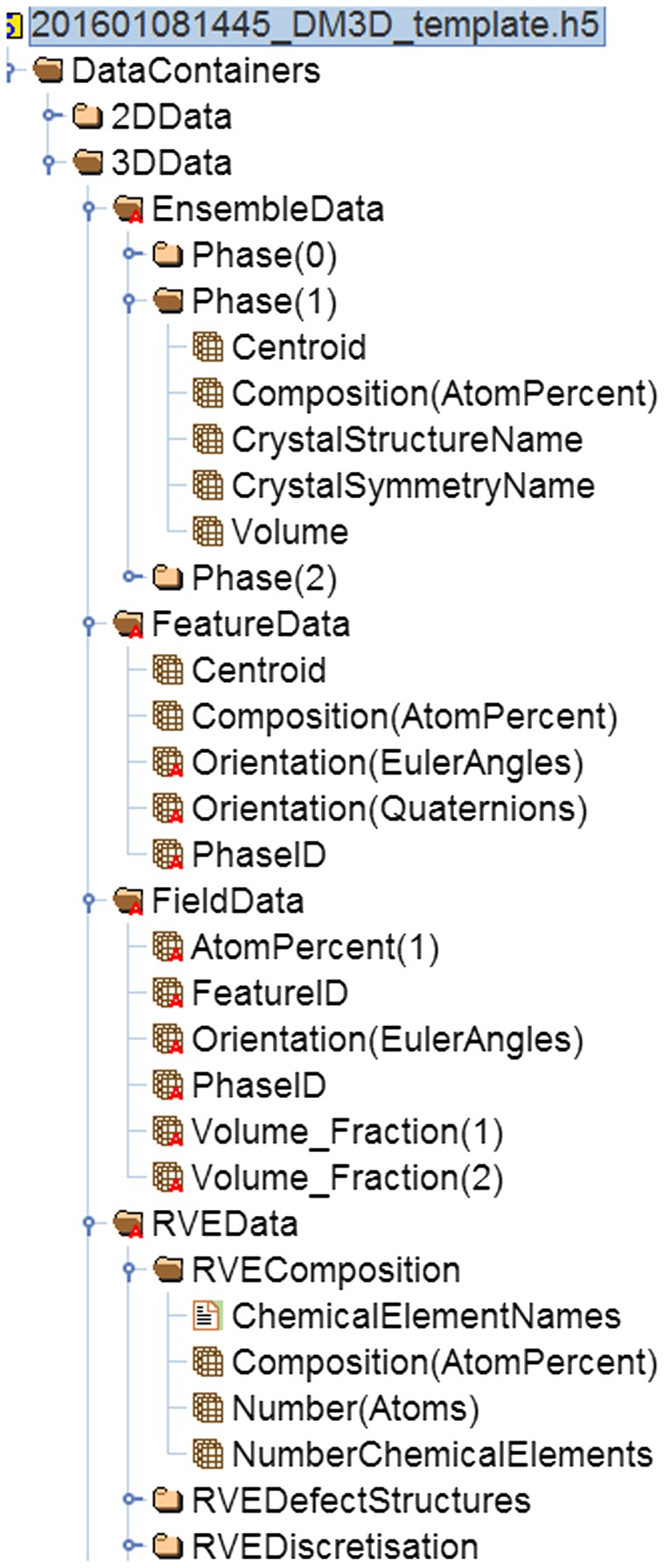
Excerpt of an HDF5 file drawing on some of the descriptors being defined in this article and their hierarchical structure. This file is based on the Al-Cu example used throughout this article. It is available as a template for download from [32] along with this publication. Visualization of the file structure proceeds e.g. by HDF5-view.[12]

This template HDF5 file (Figure 17) is available for free download from [32] and then can be inspected using e.g. HDF5-view [12] or be further exploited using tools like Dream-3D [9,10] and/or Paraview.[33,34]

The following briefly summarizes the history of the generation of this file:

• MICRESS® [32] result file comprising voxel and other data created for the simple geometry depicted throughout this paper.
• File converted to HDF5 by export functionality in MICRESS® post processor.
• Enriched by manual editing with some further descriptors defined in this paper using HDF5-view [12].
• Enriched by surface mesh creation using Dream-3D [9,10] quick surface mesh functionality.
• Enriched by mechanical properties data and objects (note the data in the objects are dummy and do NOT relate to Al-Cu!) from an Abaqus .obj file.[30,31]

## 6. Summary and conclusions

The present paper describes a comprehensive list of descriptors allowing the geometric description of any static microstructure. Numerous early efforts to generate such a list by simply collecting descriptors/keywords from a variety of different codes and trying to harmonize them into a unique set of descriptors all failed. This is essentially due to the fact that many descriptors/keywords can be derived from other descriptors/keywords and the amount of possible descriptors thus tends to infinity. Thus the need for a basic set of descriptors was identified, being minimal in their number, but allowing derivation of anything which is needed by suitable relations between the basic descriptors. The major concepts followed to generate the descriptor list depicted in the present article are:

• Starting from the most generic notion of a material as being an arrangement of atoms in space.
• Considering a dimensional hierarchy of 3D volumes, 2D surfaces, 1D lines and 0D points.
• Considering a hierarchy in terms of data resolution with the highest resolution data (the ‘*Field*’) being the basis for lower resolution and averaged data for *Features*, for *Ensembles* and eventually for statistical data of the full *RVE*.
• Proposing a scheme to derive relations between descriptors by different mathematical operations.
• Securing that the selected descriptors are independent of any numerical representation in view of interoperability with experimental data and with available analytic solutions.
• Attempting to specify descriptors which hold for both continuum and discrete models, i.e. allowing the description of discrete features in the volume and also of continuous fields.
• Attempting to specify descriptors that may even be applicable to electronic, atomistic and mesoscopic models. This has however to be carefully verified in future.

The proposed metadata structure allows assigning:

• attributes to ensembles/phases e.g. their mechanical properties;
• attributes to features like dislocation densities;
• attributes to interfaces like interfacial energies or interfacial mobilities; and
• conditions on boundaries, e.g. for temperature and/or composition, for flow velocity and others.

An outlook has been provided on further attributes which can be assigned to each of the descriptors, e.g. error estimates, mean values, data validity range, data origin, creation date and many others. The specification of unique names for such attributes and their compilation in comprehensive metadata schemata is a challenging task for the future.

A template file drawing on the proposed descriptors has been created on the basis of the HDF5 file format, which currently is discussed as a possible basis for a standardized exchange of microstructure information.[32]

The authors are well aware that the present compilation of descriptors only provides a basis to kick-start the discussions on interoperability. The presented structures will surely need future amendments and revisions. The proposed underlying conceptual approach, however, seems viable, generic and extendable. Everybody willing to volunteer is invited to contribute to the sustainability of further developments by contacting the author or any member of the ICMEg consortium.[35]

## Disclosure statement

No potential conflict of interest was reported by the authors.

## Funding

The research leading to these results has been performed within the ICMEg project and has received funding from the European Union Seventh Framework Programme [FP7/2007-2011 under grant agreement no. 6067114] and from the Cluster of Excellence ‘Integrative Production Technologies for High Wage Countries’ funded by the Deutsche Forschungsgemeinschaft.
